# Myelin Basic Protein Fragmentation by Engineered Human Proteasomes with Different Catalytic Phenotypes Revealed Direct Peptide Ligands of MS-Associated and Protective HLA Class I Molecules

**DOI:** 10.3390/ijms24032091

**Published:** 2023-01-20

**Authors:** George A. Saratov, Vasiliy I. Vladimirov, Alexey L. Novoselov, Rustam H. Ziganshin, Guo Chen, Timur N. Baymukhametov, Andrey L. Konevega, Alexey A. Belogurov, Anna A. Kudriaeva

**Affiliations:** 1 Shemyakin and Ovchinnikov Institute of Bioorganic Chemistry, Russian Academy of Sciences, 117997 Moscow, Russia; 2 Phystech School of Biological and Medical Physics, Moscow Institute of Physics and Technology (National Research University), 141701 Dolgoprudny, Russia; 3 School of Biopharmacy, China Pharmaceutical University, Nanjing 211198, China; 4 National Research Center, “Kurchatov Institute”, 123182 Moscow, Russia; 5 Institute of Biomedical Systems and Biotechnologies, Peter the Great St. Petersburg Polytechnic University, 195251 St. Petersburg, Russia; 6 Petersburg Nuclear Physics Institute Named by B.P. Konstantinov of National Research Centre, Kurchatov Institute, 188300 Gatchina, Russia; 7 Department of Biological Chemistry, Ministry of Health of Russian Federation, Evdokimov Moscow State University of Medicine and Dentistry, 127473 Moscow, Russia

**Keywords:** proteasome, HTBH, immunoproteasome, biotin carboxyl carrier domain, myelin basic protein, HLA class I, multiple sclerosis, sodium dodecyl sulfate, IFNγ, negative stain electron microscopy

## Abstract

Proteasomes exist in mammalian cells in multiple combinatorial variants due to the diverse regulatory particles and exchange of catalytic subunits. Here, using biotin carboxyl carrier domain of transcarboxylase from *Propionibacterium shermanii* fused with different proteasome subunits of catalytic and regulatory particles, we report comprehensive characterization of highly homogenous one-step purified human constitutive and immune 20S and 26S/30S proteasomes. Hydrolysis of a multiple sclerosis (MS) autoantigen, myelin basic protein (MBP), by engineered human proteasomes with different catalytic phenotypes, revealed that peptides which may be directly loaded on the HLA class I molecules are produced mainly by immunoproteasomes. We detected at least five MBP immunodominant core regions, namely, LPRHRDTGIL, SLPQKSHGR, QDENPVVHFF, KGRGLSLSRF and GYGGRASDY. All peptides, except QDENPVVHFF, which originates from the encephalitogenic MBP part, were associated with HLA I alleles considered to increase MS risk. Prediction of the affinity of HLA class I to this peptide demonstrated that MS-protective HLA-A*44 and -B*35 molecules are high-affinity binders, whereas MS-associated HLA-A*23, -A*24, -A*26 and -B*51 molecules tend to have moderate to low affinity. The HLA-A*44 molecules may bind QDENPVVHFF and its deamidated form in several registers with unprecedently high affinity, probably linking its distinct protective phenotype with thymic depletion of the repertoire of autoreactive cytotoxic T cells or induction of CD8+ regulatory T cells, specific to the encephalitogenic MBP peptide.

## 1. Introduction

The ubiquitin–proteasome system and the autophagy–lysosome system are essential intracellular machinery [1,2]. The proteasome is assembled from a catalytic core particle, also termed the 20S proteasome, which is capped by different regulatory particles. Three types of hydrolytic activities, namely, chymotryptic, tryptic and caspase-like, are provided by catalytic subunits of the 20S particle. The catalytic particle may exist in two forms, termed constitutive and immunoproteasome. The last one bears catalytic subunits with reduced caspase activity and a more chymotryptic phenotype. Regulatory particles are broadly diversified and may bind two ends of the 20S independently, forming so-called hybrid proteasomes. Summarizing, proteasomes may exist in the cell in the multiple combinatorial variants, which are functionally different in terms of the composition, substrate engaging and protein degradation pattern [1]. One of the major functions of the proteasome is to generate peptides which are then presented on cell surface in the context of the HLA class I molecules. These peptide patterns form “immune passport” of the cell, which is a basis of the anti-tumor [3] and anti-viral defense [4]. Unfortunately, the same evolutionarily perfect system of the CD8+ T cell-mediated cytotoxicity is involved in the pathogenesis of the autoimmune disorders [5], including chronic demyelinating syndrome—multiple sclerosis (MS) [6,7].

Multiple sclerosis is one of the most common autoimmune diseases. An inflammatory reaction in the human central nervous system leads to the axonal damage, demyelination and neurodegeneration, which involve the proteolytic degradation of myelin basic protein (MBP) [8,9]. Despite many years of study of this disease, its etiology is still debated, and methods of treatment are quite limited [10]. Deregulation of the ubiquitin-proteasome system is known to induce broad changes in human homeostasis, including neurodegenerative and autoimmune diseases [11]. Our recent studies demonstrated that one of the key autoantigens in MS, myelin basic protein, is hydrolyzed by proteasome without ubiquitination [12,13] due to a novel class of the charge-mediated proteasomal degrons [14,15,16]. Thus, ubiquitination system generally fails to control MBP entrapment by the proteasome, which leads to recognition of the oligodendrocytes by myelin-reactive cytotoxic lymphocytes [17]. Here, using engineered human proteasomes with different catalytic phenotypes, we elucidate how MBP may be processed in the inflammation sites of the central nervous system during MS pathogenesis.

## 2. Results

### 2.1. Purification and Characterization of the HTBH-Engineered Proteasomes

**Figure 1:**
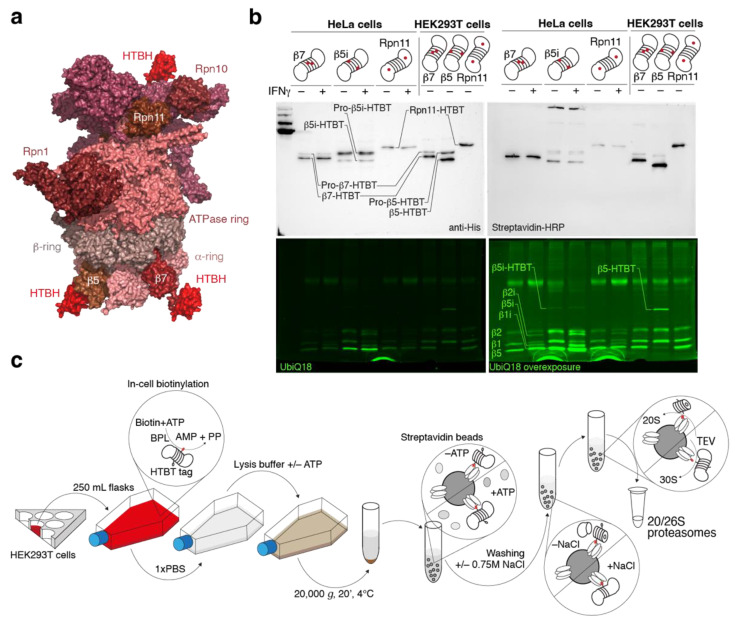
Engineered human proteasomes. (**a**) Model of the 26S proteasome (5GJR) and biotin carboxyl carrier domain of the transcarboxylase from *Propionibacterium shermanii* (1DCZ) fused with β5 (PSMB5), β7 (PSMB4) and Rpn11 (PSMD14). (**b**) Analysis of the HEK293 cell lysates utilizing anti-His antibodies, streaptavidin-HRP and a Me_4_BodipyFL-Ahx_3_Leu_3_VS fluorescent probe (UbiQ18). (**c**) Purification of the HTBH-tagged proteasomes from mammalian cells.

The DNA constructs coding for HTBH-tagged [18,19,20] proteasome subunits β5 (PSMB5), b5i (PSMB8), β7 (PSMB4) and Rpn11 (PSMD14) (Figure 1a) were integrated into the genomes of HEK293T and HeLa cells using the Sleeping Beauty transposon system [21]. The β5 and β5i subunits are responsible for the chymotrypsin-like activity of the constitutive proteasome and immunoproteasome, respectively. Proteasome purification from HEK293T and HeLa cells with overexpressed β5i and β5, respectively, resulted in extremely low yields of the complex, and these variants were excluded from further experiments (data not shown). β7 is a non-catalytic subunit of the 20S inner β-ring, and Rpn11 is a subunit of the 19S regulatory particle with deubiquitinase activity. The HTBH tag consists of the tobacco etch virus nuclear-inclusion-a endopeptidase (TEV) site and the biotin carboxyl carrier domain of transcarboxylase from *Propionibacterium shermanii*, flanked with two 6-histidine clusters.

**Figure 2:**
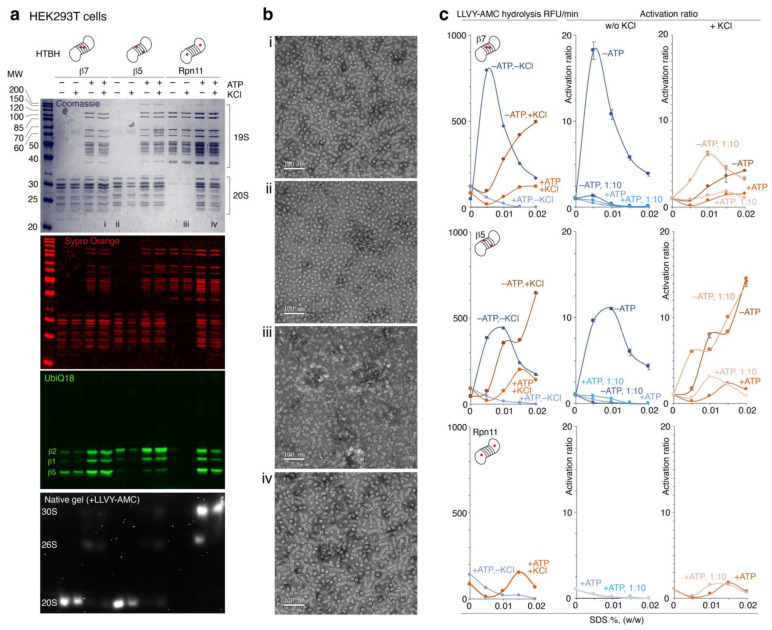
Activation profile of the engineered proteasomes isolated from HEK293T cells in the presence of SDS. (**a**) Analysis of the proteasomes purified with or without ATP and eluted from the streptavidin beads in the presence or absence of the 100 mM KCl. From top to bottom: SDS-PAGE stained Coomassie and Sypro Orange, in-gel fluorescence staining using Me_4_BodipyFL-Ahx_3_Leu_3_VS fluorescent proteasome probe (UbiQ18), native PAGE with LLVY-AMC substrate. (**b**) Representative images of the negative staining electron microscopy of the proteasome samples, as indicated on the Coomassie-stained gel in panel (**a**). (**c**) SDS activation profile of the purified proteasomes eluted with (orange curves) or without (blue curves) 100 mM KCl. Note: 1:10 means ten-times diluted proteasome. Data are shown as averages with standard deviations.

Mammalian cells stably expressing HTBH-tagged proteasome subunits (Figure 1b) were lysed in the presence or absence of ATP, and further protein lysates were incubated with streptavidin beads (Figure 1c). Washing buffer for samples without ATP was supplemented with 0.75 M NaCl to dissociate 20S and 19S particles. Purified proteasomes were analyzed by gel electrophoresis utilizing Coomassie and Sypro Orange staining, in-gel fluorescence with Me_4_BodipyFL-Ahx_3_Leu_3_VS fluorescent proteasome probe (UbiQ18) [22] and native gel (Figure 2a).

The 20S and 26S proteasomes (Figure 2a,b) purified from HEK293T cells were analyzed in terms of activation by sodium dodecyl sulfate (SDS) in the presence or absence of the 100 mM KCl (Figure 2c). The 20S proteasomes isolated via HTBH-tagged β5 and β7 subunits had similar activation profiles with maxima at SDS concentrations of 0.005–0.01%. The presence of the 100 mM KCl in the reaction buffer resulted in an unsaturated activation curve. Moreover, in the presence of 100 mM KCl, dilution of 20S samples resulted in an enhanced activation rate.

**Figure 3:**
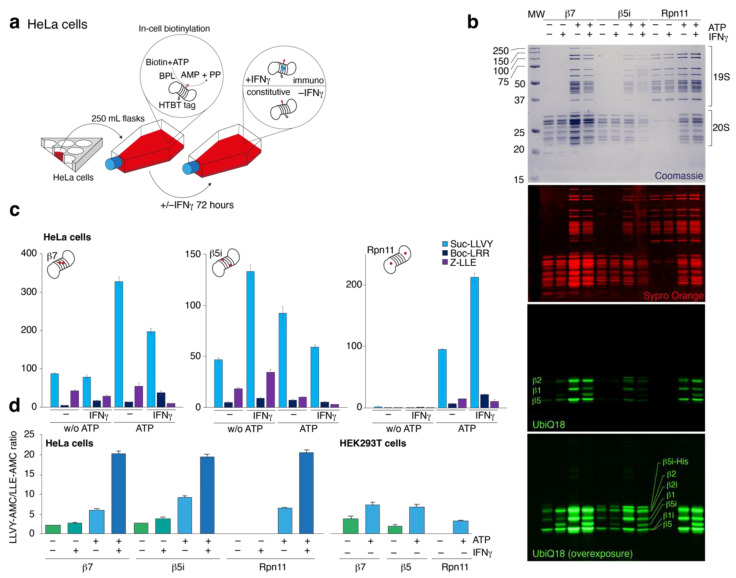
Analysis of chymotryptic, tryptic and caspase-like activities of proteasomes isolated from HeLa cells. (**a**) HeLa cells were grown in the presence or absence of IFNγ for 72 h. (**b**) Analysis of the proteasomes purified with or without ATP from cells exposed or not exposed to IFNγ. From top to bottom: SDS-PAGE stained Coomassie and Sypro Orange, in-gel fluorescence staining using Me_4_BodipyFL-Ahx_3_Leu_3_VS fluorescent proteasome probe (UbiQ18). (**c**) Analysis of chymotryptic (blue), tryptic (deep blue) and caspase-like (violet) activities of proteasomes isolated from HeLa cells. (**d**) Ratio of chymotryptic to caspase activity of proteasomes, isolated from HeLa cells and HEK293T cells in the absence of ATP not exposed (green) and exposed to IFNγ (aquamarine) or in the presence of ATP not exposed (blue) and exposed to IFNγ (deep blue). Data are shown as averages and standard deviations.

The HeLa cells were exposed to 400 U/mL of IFNγ for 72 h to induce expression of the proteasome catalytic immunosubunits (Figure 3a,b). Ratio of chymotryptic to caspase activity of 26S proteasomes, isolated from cells exposed to IFNγ, increased approximately three times in comparison with non-treated cells (Figure 3c). Despite a similar immunophenotype of the 20S and 26S proteasomes from IFNγ-treated HeLa cells according to fluorescent proteasome probe (Figure 3b), the chymotryptic to caspase activity ratio of the 20S proteasomes increased only 20–30% upon IFNγ addition (Figure 3d).

### 2.2. Fragmentation of Myelin Basic Protein by Proteasomes with Different Catalytic Phenotypes

**Figure 4:**
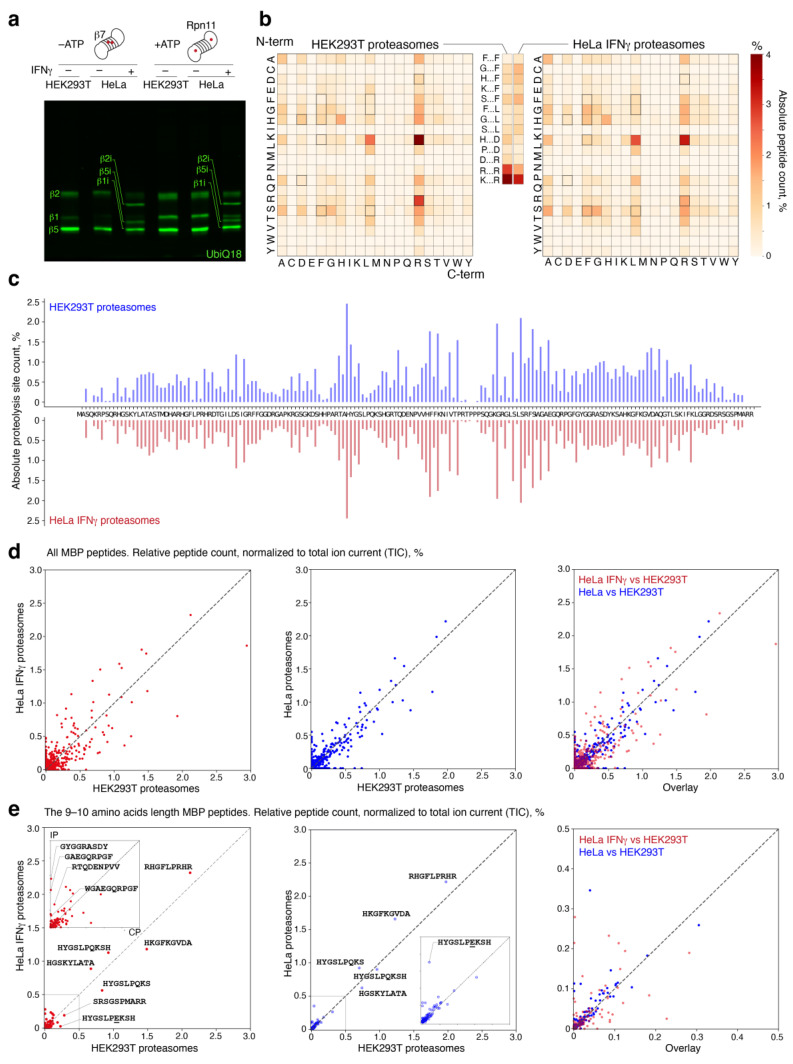
MBP hydrolysis by human-engineered proteasomes with different catalytic phenotypes. (**a**) Analysis of the proteasomes purified with or without ATP from cells exposed or not exposed to IFNγ, in-gel fluorescence staining using Me4BodipyFL-Ahx3Leu3VS (UbiQ18) fluorescent proteasome probe. (**b**) N- and C-terminal amino acids of peptides generated by proteasomes purified from HEK293T cells and HeLa cells exposed to IFNγ during MBP hydrolysis. The numbers of peptides with the corresponding combinations of first and last amino acids were counted and normalized to the total number of MBP peptides. (**c**) Fragmentation of MBP (P02686-5 isomer) by proteasomes from HEK293T cells and HeLa cells exposed to IFNγ. The hydrolysis sites were counted and normalized to the total number of hydrolysis events. (**d**) All MBP peptides generated by proteasomes purified from HeLa cells exposed to IFNγ versus HEK293T proteasomes (**left plot**) and proteasomes isolated from HeLa cells not exposed to IFNγ and HEK293T cells (**middle plot**); overlap (**right**). (**e**) The 9–10-amino-acid MBP peptides generated by proteasomes purified from cells exposed to IFNγ versus HEK293T proteasomes (**left plot**), proteasomes isolated from HeLa cells not exposed to IFNγ and HEK293T cells (**middle plot**) and overlap (**right**). Inserts represent the axis scale in the 0–0.5% range. Underlined amino acid represents Q to E deamidation. Each dot represents an individual peptide. Relative peptide count was normalized to total ion current (TIC).

In order to obtain mostly homogenous immunoproteasomes, we increased exposure of HeLa cells to IFNγ to up to 96 h and further isolated proteasomes with different catalytic phenotypes from HEK293T, HeLa and HeLa cells subjected to IFNγ treatment (Figure 4a). As HeLa cells exposed to IFNγ still contain constitutive proteasomes, this sample represented a mixture of immuno- and constitutive proteasomes, predominantly containing immunoproteasomes. Further, myelin basic protein (MBP) was incubated with purified proteasomes, and hydrolyzates were next analyzed using LS-MS/MS (Figure 4b–e). As we previously showed, MBP can be hydrolyzed by both 26S and 20S proteasomes, at least in vitro [12,14]. Although the 20S subparticle is an integral part of the 26S holoenzyme, it is also quite abundant as a free complex in many cell types [23], especially under stress conditions [24]. Thus, we analyzed equimolar mixture of MBP hydrolysates generated by 20S and 26S proteasomes from HEK293T cells, HeLa cells and HeLa cells exposed to IFNγ. Proteasomes purified from HeLa cells exposed to IFNγ, as anticipated, demonstrated more chymotryptic and less caspase and tryptic phenotype (Figure 4b). All proteasomes revealed more sites in the middle part of the MBP sequence (Figure 4c), as judged by the absolute peptide count. Pairwise comparison showed similar patterns for proteasomes from HEK293T and HeLa cells, whereas proteasomes isolated from HeLa cells exposed to IFNγ demonstrated a significant change in spectrum of the generated MBP peptides (Figure 4d). Detailed analysis of 9–10-amino-acid peptides generated by proteasomes purified from HeLa cells exposed to IFNγ- and HEK293T proteasomes revealed a significant increase in the amount of immunodominant peptides upon MBP hydrolysis by proteasomes purified from HeLa cells exposed to IFNγ (Figure 4e).

## 3. Discussion

We obtained highly homogenous human 20S and 26S constitutive- and immunoproteasomes from HEK293T and HeLa cells constitutively expressing various proteasome subunits with the HTBH tag. Our data suggest that proteasomes should be maintained in buffers of low anionic strength, as salt likely leads to aggregation of the 20S particles and partial dissociation of the regulatory particles from the 26S proteasomes. The proteasome samples were used for fragmentation of MBP, the MS autoantigen, to elucidate what MBP peptides may be presented by molecules encoded by human HLA class I alleles.

**Figure 5:**
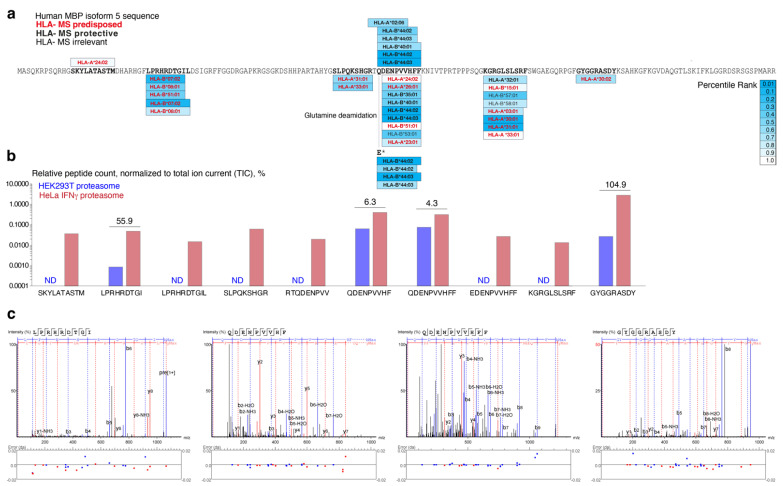
(**a**) MBP (P02686-5 isomer) sequence with marked peptides (bold), which may be presented by MS-associated (bold red) and protective (bold black) HLA class I molecules. Calculated percentile rank is shown by a gradient from white to blue (0.01–1). (**b**) Relative peptide count, normalized to total ion current, observed in the MBP hydrolyzates by HEK293T proteasomes (blue bars) and proteasomes purified from HeLa cells exposed to IFNγ (red bars). Ratio between values is shown, ND—not detected. (**c**) The MS2 spectra of analyzed MBP peptides.

Prediction of the affinity of the MBP peptides released by HEK293T proteasomes and proteasomes purified from HeLa cells exposed to IFNγ revealed an increased frequency of the immunoproteasome-generated 9–10-amino-acid peptides, which may be directly loaded on the HLA class I molecules (Figure 5, Table S1). MS-associated HLA class I molecules, which may directly bind MBP peptides, mostly produced by the immunoproteasome, were encoded by HLA-A*03, -A*23, -A*24, -A*26, -A*30, -A*31, -A*33, -B*07, -B*08, -B*15 and -B*51 alleles (Figure 5a).

The product of HLA-A*30:02 allele, which is more frequent in Bahrain MS patients compared with in control subjects [25], is capable of binding the MBP peptide GYGGRASDY, which is released by proteasomes purified from cells exposed to IFNγ two orders of magnitude more efficiently than HEK293T proteasomes (Figure 5b). MBP peptide KGRGLSLSR, generated solely by proteasomes purified from HeLa cells exposed to IFNγ (Figure 5b), is a potential high-affinity ligand for molecules encoded by HLA-A*30:01 and HLA-A31:01 alleles (Figure 5a). The same peptide may be bound by a product of the HLA-A*03:01 allele (Figure 5a), which is more frequent in Iranian patients [26]. The HLA-A*03:01 molecule also binds the KLIETYFSK peptide—a naturally processed epitope of the proteolipid protein (PLP) [27], which is similar to MBP peptide in terms of the flanking basic residues. Thus, one distinct HLA class I molecule may present peptides from both major MS autoantigens. Interestingly, Rajaei et al. showed that in MS patients, the HLA-A*31 allele was often in combination with HLA-A*03 and HLA-A*24 [28]. We thus suggest that synergetic presentation of myelin peptides by heterozygote HLA-A-encoded alleles may significantly increase the risk of MS development.

The HLA-B*07:02, -B*08:01 and -B*51:01 molecules may bind the MBP peptide LPRHRDTGIL with moderately high affinity (Figure 5a). The amount of this peptide was increased 50-fold in MBP hydrolysates mediated by proteasomes purified from HeLa cells exposed to IFNγ than with HEK293T proteasomes (Figure 5b). Jilek et al. showed that the HLA-B*07:02-restricted EBV-specific (epitope spanning residues 379–387 of EBNA-3, EBVRPP, RPPIFIRRL) CD8+ T cell response is dysregulated in MS patients [29]. One may suggest that the ability of molecules encoded by the HLA-B*07:02 allele to present MBP and EBNA-3 epitopes may be additional evidence confirming theory of molecular mimicry, linking EBV and MS [30,31]. These findings are especially important in terms of the recently reported association of the EBV and MS [32].

Among MS-protective HLA alleles, we found that HLA-B*44 molecules may bind MBP peptide QDENPVVHFF, whereas HLA-A*02:06-encoded molecule may bind RTQDENPVV (Figure 5a). Interestingly, both peptides span the classical MBP immunodominant epitope [33]. Healy et al. reported that HLA-A*02 and HLA B*44 alleles both reduce susceptibility to MS; however, only HLA-B*44 seems to influence disease course, preserving brain volume and reducing the burden of T2 hyperintense lesions in subjects with MS [34]. On the other hand, we showed that core peptide DENPVVHFF also may be a potential ligand of MS-associated HLA-A*23:01, HLA-A*24:02, HLA-A*26:01 and HLA-B*51:01-encoded molecules (Figure 5a). The frequencies of HLA-A*23 and -A*26 alleles were significantly higher in MS patients than in controls in Italian population [35]. The HLA-B*51 was more frequent in patients with pars planitis [36], often accompanying MS [37].

Here we demonstrated that the predicted affinity of HLA class I to the core MBP peptide QDENPVVHFF of MS-associated HLA-A*23, -A*24, -A*26 and -B*51 molecules tends to be moderate to low (Figure 5a). In contrast, molecules encoded by protective HLA-A*44 alleles may bind this peptide, and its deamidated form, in several registers with unprecedently high affinity (percentile rank = 0.01) (Figure 5a). Further studies should elucidate if the ability of HLA-B*44 to efficiently present an encephalitogenic MBP peptide may affect MS’s course, probably due to the exhaustive thymic negative selection of myelin-reactive—or induction of regulatory MBP-specific—CD8+ T cells [38,39].

Proteasome inhibitors may be of interest as therapeutic agents for MS treatment. Some inhibitors, such as bortezomib (PS-341), have been tested as a treatment for MS in experimental autoimmune encephalomyelitis (EAE), which serves as the animal model of MS. These experiments have resulted in a decrease in the number of T cells that secrete pro-inflammatory cytokines [11]. Neurodegeneration is also characterized by activation of the immunoproteasome to the detriment of the constitutive proteasome, 202 [17,40,41]. Therefore, the development of inhibitors specific to proteasome immunosubunits may represent new therapeutic approaches to various forms of MS and other neurodegenerative and neuro-immunological disorders, especially for individuals carrying HLA class I MS-risk alleles.

## 4. Materials and Methods

### 4.1. Mammalian Cells Constitutively Expressing HTBH-Tagged Proteasomes

HEK293T (human embryonic kidney) and HeLa (human, cervical adenocarcinoma) cells were obtained from shared research facility "Vertebrate cell culture collection" of the Institute of Cytology Russian Academy of Sciences. All mammalian cell lines were cultured in DMEM medium (GIBCO, Thermo Fisher Scientific, Waltham, MA, USA). The media was supplemented with 10% fetal bovine serum (GIBCO, Thermo Fisher Scientific, Waltham, MA, USA) and 1% antibiotic-antimycotic (GIBCO, Thermo Fisher Scientific, Waltham, MA, USA). The cell lines were incubated in 37 °C humidified incubator with 5% CO_2_. All cells were routinely tested for Mycoplasma contaminations.

The Sleeping beauty transposon system was used to generate HEK293T and Hela cells that overexpressed human PSMB4, PSMB5, PSMB8 and PSMD14 proteasome subunits with HTBH tag. Cells were co-transfected with Sleeping Beauty transposon plasmid pSBi-Pur (PSMB4-HTBH, PSMB5-HTBH, PSMB8-HTBH or PSMD14-HTBH cDNA) and Sleeping Beauty transposase plasmid pCMV (CAT) T7-SB100 in ratio 10:1 with Lipofectamine 3000 (Thermo Fisher Scientific, Waltham, MA, USA). Three days after transfection, cells were maintained in selection medium (1 μg/mL puromycin in DMEM growth medium) for at least seven days.

Sleeping Beauty transposon vector pSBbi-Pur (Addgene no.60523) and the pCMV(CAT)T7-SB100 (Addgene no. 34879) containing the cytomegalovirus (CMV) promoter and SB100X transposase were gifts to Addgene from Eric Kowarz and Zsuzsanna Izsvak, respectively. The sequences encoding the PSMB4, PSMB5, PSMB8 and PSMD14 proteasome subunits were amplified from cDNA isolated from Hela cells via PCR, then overlapped with HTBH tag sequence and subcloned into pSBbi-Pur vector.

### 4.2. Purification of the HTBH-Tagged Proteasomes

Stable HeLa or HEK293T cell lines expressing HTBH-tagged proteasomes were grown to confluence and then washed with PBS buffer. The cell pellets were lysed in buffer (30 mM Tris-HCl pH 7.5, 5 mM MgCl_2_, 0.5% NP-40, 1 mM TCEP, 1 mM PMSF, +/−100mM KCl and 5 mM ATP (ATP was added only to purify 26S/30S proteasomes)). The lysates were centrifuged at 20,000× *g* for 15 min to remove cell debris, and the supernatant was incubated with Streptavidin–agarose (Thermo Fisher Scientific, Waltham, MA, USA) resin overnight at 4 °C with constant rotation. To purify the 20S proteasome, the streptavidin beads were washed 2 times with 10 volumes of the buffer (30 mM Tris-HCl pH 7.5, 5 mM MgCl_2_, 1 mM TCEP, +/−100 mM KCl, 750 mM NaCl), followed by another 2 washes with buffer without 750 mM NaCl. To purify the 26S proteasome, the streptavidin beads were washed 4 times with 10 volumes of the buffer (30 mM Tris-HCl pH 7.5, 5 mM MgCl_2_, 1 mM TCEP, +/−100 mM KCl and 1 mM ATP). Next, the beads were resuspended in the required volume of wash buffer with TEV protease, His (Genscript Biotech, Piscataway, NJ, USA) and were incubated at 30 °C for 2 h.

### 4.3. Analysis of the Proteasome Activity

The peptidase activity was determined with 0.125 μg proteasome incubated with 20 μM of the fluorogenic substrate Suc-LLVY-AMC, Boc-LRR-AMC, or Z-LLE-AMC (excitation wavelength of 380 nm and an emission wavelength of 440 nm) in a volume of 25 μL by a microplate reader (CLARIOstar plus, BMG Labtech, Ortenberg, Germany) at 37 °C. The buffer used for measurement of the activity of the proteasomes contained 30 mM Tris-HCl pH 7.5, 5 mM MgCl_2_, 1 mM TCEP and 1 mM ATP with various SDS concentrations. Data related to analysis of inhibition of purified proteasome samples by specific inhibitor MG132 are presented in Figure S1.

### 4.4. In-Gel Fluorescence with Me_4_BodipyFL-Ahx_3_Leu_3_VS Fluorescent Proteasome Probe

Equal amounts of protein were incubated with Me4BodipyFL-Ahx3Leu 3VS for 30 min at 37 °C in buffer (30 mM Tris-HCl pH 7.5, 5 mM MgCl_2_, 1 mM TCEP, +/−100 mM KCl, and 1 mM ATP (for 26S/30S proteasomes)). This was followed by adding sample buffer containing β-mercaptoethanol. Samples were analyzed using 12% sodium dodecyl sulfate polyacrylamide gel electrophoresis (SDS-PAGE). Wet gel slabs were imaged using the imaging system (ChemiDoc, Bio-Rad, Hercules, CA, USA) with appropriate filter settings (λ(ex/em) = 480/530 nm). To verify protein loading, gels were stained with a SYPRO™ Orange Protein Gel Stain and imaged using appropriate filter settings (λ(ex/em) = 470/570 nm). Alternatively, protein loading was verified by staining gels with a Coomassie blue stain.

### 4.5. Native PAGE with LLVY-AMC

All samples were analyzed on mini gels using a Mini-Protean gel apparatus (Bio-Rad, Hercules, CA, USA). Proteasome samples were resolved by nondenaturing PAGE by a modified version of the method described in [42]. We used a gel consisting of 0.09 M Tris (pH 8.3), 0.09 M H_3_BO_3_, 5 mM MgCl_2_, 1 mM ATP, 1 mM DTT and acrylamide-bisacrylamide at a ratio of 37.5:1 (4% for resolving gel and 2.5% for stacking gel) and polymerized with 0.1% N,N,N′,N′-tetramethylethylenediamine (TEMED) and 0.1% ammonium persulfate. The running buffer was the same as the gel buffer but without acrylamide. Nondenaturing minigels were run at 150 V for approximately 2 h at +4 °C. The gels were then incubated in 10 mL of 0.1 mM Suc-LLVY-AMC in activity assay buffer (20 mM Tris-HCl pH 7.5, 5 mM MgCl_2_, 1 mM ATP, 1 mM DTT) for 30 min at 37 °C. Proteasome bands were visualized upon exposure to UV light (360 nm) and photographed using the imaging system (ChemiDoc, Bio-Rad, Hercules, CA, USA).

### 4.6. Negative Staining Electron Microscopy

For negative staining, 3 µL of the purified proteasome samples was applied to glow-discharged EM grids covered by a thin layer of continuous carbon film (0183-F, Ted Pella Inc., Redding, CA, USA). After a 1 min incubation, the grid was “washed off” at an angle with 25 μL of 2% uranyl-acetate solution. After 1 min, excess uranyl-acetate was blotted, and the grid was dried. Observations of the sample were performed with a Titan 80–300 transmition electron microscope (Thermo Fisher Scientific, Waltham, MA, USA).

### 4.7. MBP Hydrolisis by Proteasomes

The hydrolysis of human myelin basic protein (2 μg) was performed in a volume of 20 μL. Reaction buffer for 20S proteasomes (0.2 μg) contained 20 mM Tris (pH 7.5), 5 mM MgCl_2_ and 1 mM TCEP. Reaction buffer for 26S proteasomes (0.2 μg) contained 20 mM Tris (pH 7.5), 5 mM MgCl_2_, 1 mM ATP and 1 mM TCEP. The mixtures were incubated for 2 h at 37 °C.

### 4.8. Mass Spectrometry

Samples were loaded into an in-house-made trap column 50 × 0.1 mm, packed with Prontosil 120-C18AQ 5 μm sorbent (Dr. Maisch, Ammerbuch, Germany), in the loading buffer (2% ACN, 98% H_2_O, 0.1% TFA) at 4 μL/min flow and separated at RT in an in-house-packed [43] fused-silica column 300 × 0.1 mm packed with Reprosil PUR C18AQ 1.9 (Dr. Maisch, Ammerbuch, Germany) into an emitter prepared with P2000 Laser Puller (Sutter Instrument, Novato, CA, USA). Reverse-phase chromatography was performed with an Ultimate 3000 Nano LC System (Thermo Fisher Scientific, Waltham, MA, USA), which was coupled to the Q Exactive Plus Orbitrap mass spectrometer (Thermo Fisher Scientific, Waltham, MA, USA) via a nanoelectrospray source (Thermo Fisher Scientific, Waltham, MA, USA). Peptides were loaded in a loading solution (98% 0.1% (*v*/*v*) formic acid, 2% (*v*/*v*) acetonitrile) and eluted with a linear gradient: 3–6% B for 3 min; 6–25% B for 30 min, 25–40% B for 25 min, 40–60% B for 4 min, 60% B during 3 min, 60–99% B for 0.1 min, 99% B during 10 min, 99–2% B for 0.1 min at a flow rate of 500 nL/min. MS1 parameters were as follows: 70 K resolution, 350–1600 scan range, maximum injection time—35 msec, AGC target—3 × 10^6^. Ions were isolated with a 1.4 *m*/*z* window, preferred peptide match and isotope exclusion. Dynamic exclusion was set to 30 s. MS2 fragmentation was carried out in HCD mode at 17.5 K resolution with HCD collision energy 30%, maximum injection time—80 msec, AGC target—1 × 10^5^. Other settings: charge exclusion—unassigned, 1, >7.

MS raw files were analyzed by Peaks studio 10.0 (Bioinformatics Solutions Inc., Waterloo, ON, Canada). Identification of proteins was performed by searching against the Homo sapiens Uniprot FASTA database, 9 July 2021 version, with carbamidomethyl Cys as a fixed modification and deamidation of Asn/Gln and Met oxidation as variable modifications. The false-discovery rate for peptide-spectrum matches was determined by searching a reverse database and was set to 0.01. Enzyme specificity was set as cleavage after arginine and lysine amino acid residues, and a maximum of two missed cleavages were allowed in the database search. Peptide identification was performed with an allowed initial precursor mass deviation up to 10 ppm and an allowed fragment mass deviation 0.05 Da.

### 4.9. Bioinformatics

The proteome data were analyzed within the in-house algorithm. Briefly, specific MBP peptides were separated out from other irrelevant peptides. The specific MBP peptide count was normalized to total MBP ion current (TIC). Overall, we identified roughly 18,000 specific MBP peptides (18,216), which included both modified and non-modified peptides. HLA affinity binding of the peptides was predicted by IEDB API Tools Python SDK (application iedb 0.0.2, https://pypi.org/project/iedb/, accessed on 11 May 2021), with recommended default settings [44]. The artificial neural network model returns a likelihood of a peptide being a natural ligand of the selected MHC(s). With this approach, we estimated binding affinity for peptides 9–10 amino acids in length versus 27 HLA clas I alleles.

## Data Availability

The mass spectrometry proteomics data have been deposited into the ProteomeXchange Consortium via the PRIDE partner repository with the dataset identifier PXD038387 and 10.6019/PXD038387.

## Supplementary Materials

The following supporting information can be downloaded at: https://www.mdpi.com/article/10.3390/ijms24032091/s1. Figure S1. Analysis of inhibition of purified proteasome samples by MG132. Table S1 (MBP_Peptides_CP_IP). MBP peptides generated by HEK293T proteasomes and proteasomes purified from HeLa cells exposed to IFNγ. Sheet 1 (Total MBP peptides) contains the columns with peptide sequence, peptide length, average relative count for the peptide in MBP hydrolyzates by HEK293T proteasomes (Constitutive), proteasomes purified from HeLa cells exposed to IFNγ (Immuno), the ratio of HEK293T proteasomes versus proteasomes purified from HeLa cells exposed to IFNγ (Constitutive/Immuno) and proteasomes purified from HeLa cells exposed to IFNγ versus HEK293T proteasomes (Immuno/Constitutive). Sheet 2 (HLA class I ligands) contains prediction of peptides binding to HLA class I molecules. The columns contain the HLA class I allele, the input sequence number (#), start position and end position of the peptide, its length, the peptide sequence, the predicted score of binding to HLA and percentile rank, average relative count for the peptide in MBP hydrolyzates by HEK293T proteasome (Const), proteasomes purified from HeLa cells exposed to IFNγ (Immuno) and IFNγ’s ratio (IP/CP ratio).
