# Supplementary material for: Myelin Basic Protein Fragmentation by Engineered Human Proteasomes with Different Catalytic Phenotypes Revealed Direct Peptide Ligands of MS-Associated and Protective HLA Class I Molecules
Source: Int J Mol Sci. 2023 Jan 20;24(3):2091. doi: 10.3390/ijms24032091 (PMC9917034; doi:10.3390/ijms24032091)
Supplement: Supplementary file 1 [file ijms-24-02091-s001.zip › Figure S1.pdf]

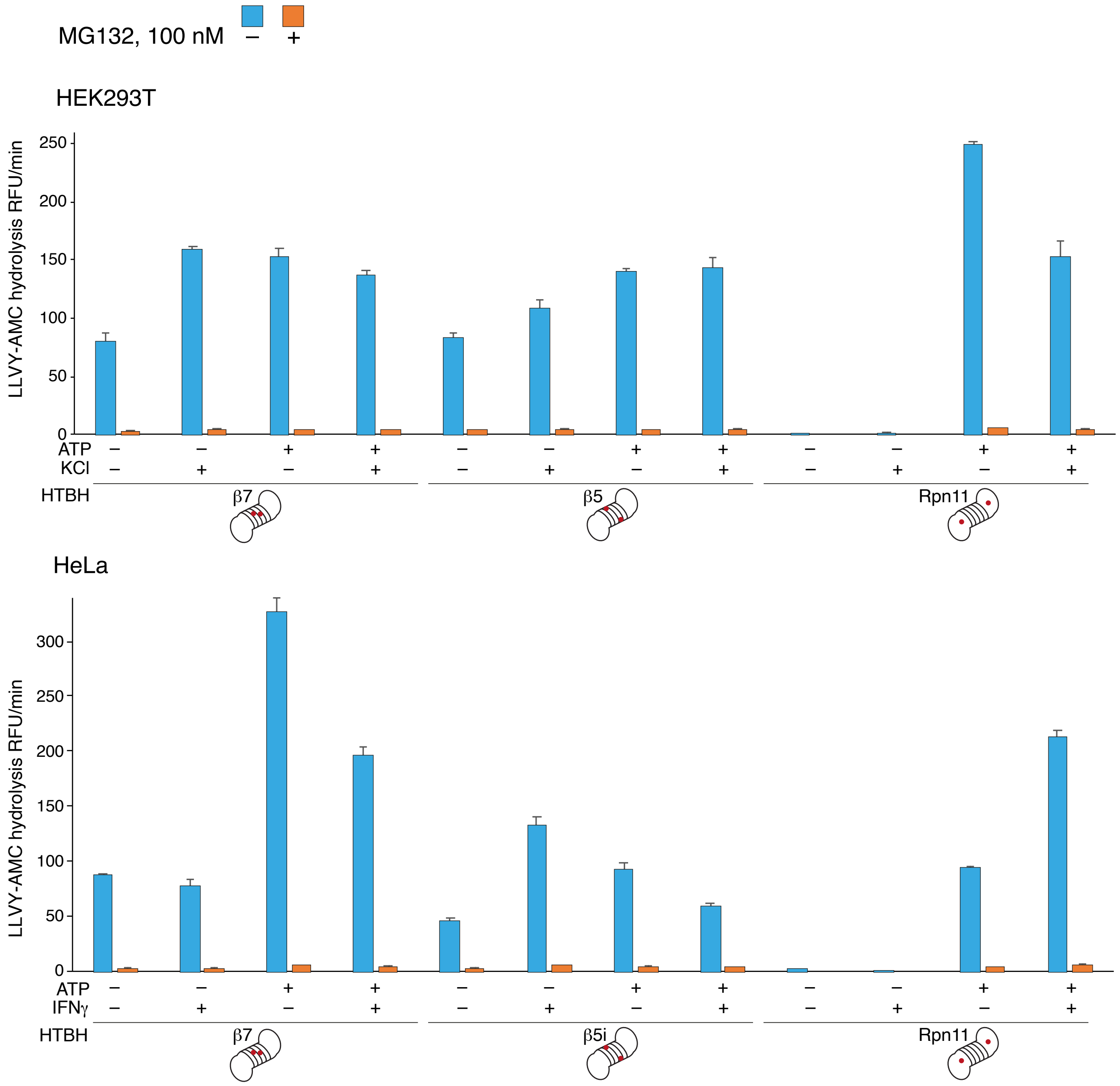

Supplementary Figure S1. Analysis of chymotryptic activities of proteasomes isolated from HEK293T and HeLa cells in the absence (blue) or presence (orange) of 100 nM of proteasome inhibitor MG132.
